# Non-alcoholic fatty liver disease and metabolic syndrome in Brazilian middle-aged and older adults

**DOI:** 10.1590/S1516-31802007000600006

**Published:** 2007-11-01

**Authors:** Mauro Karnikowski, Cláudio Córdova, Ricardo Jacó de Oliveira, Margô Gomes de Oliveira Karnikowski, Otávio de Tolêdo Nóbrega

**Affiliations:** Stricto sensu postgraduate program in Gerontology, Universidade Católica de Brasília (UCB), Taguatinga, Federal District, Brazil

**Keywords:** Fatty liver, Aged, Middle aged, Metabolic syndrome X, Brazil, Esteatose hepática, Idoso, Meia-idade, Síndrome x metabólica, Brasil

## Abstract

**CONTEXT AND OBJECTIVES:**

Non-alcoholic fatty liver disease (NAFLD) is a complex clinicopathological entity characterized by diffuse or focal fat accumulation in the hepatic parenchyma of patients who deny abusive alcohol consumption. This study aimed to assess idiopathic NAFLD in community-dwelling, middle-aged and older adults living in the Brazilian Federal District. Associations between NAFLD and components of metabolic syndrome and the whole syndrome were investigated.

**DESIGN AND SETTINGS:**

This was a cross-sectional study on 139 subjects aged 55 years or older.

**METHODS:**

NAFLD was diagnosed by means of clinical procedures, to exclude subjects with signs of liver disorders, abusive alcohol consumption and influence from hepatotoxic drugs. Phenotypes were graded based on ultrasound examination. Metabolic syndrome was defined using the NCEP ATP III criteria. Laboratory tests were performed to assist clinical examinations and define the syndrome.

**RESULTS:**

NAFLD was present in 35.2% of the subjects. Taken together, the two most intense phenotypes correlated with increased serum fasting glucose, triglyceride and VLDL cholesterol levels. Metabolic syndrome was diagnosed in 25.9% of the sample. In addition to associating NAFLD with specific traits of metabolic syndrome, non-parametric analysis confirmed the existence of a relationship (p < 0.05) between the steatotic manifestation and the syndromic condition.

**CONCLUSION:**

Compared with the literature, this study reveals greater frequency of idiopathic NAFLD among Brazilian middle-aged and older adults than is described elsewhere. The findings also suggest that impaired glycemic metabolism coupled with increased fat delivery and/or sustained endogenous biosynthesis is the most likely physiopathogenic mechanisms underlying the onset of NAFLD in this population.

## RESUMEN

**contexto E OBJETIVOS:**

Esteatose hepática não-alcoólica (EHNA) constitui entidade clínico-patológica caracterizada por acúmulo focal ou difuso de gordura no parênquima hepático de pacientes que negam consumo abusivo de álcool. Este estudo avaliou a freqüência de EHNA entre indivíduos idosos e de meia-idade residentes em comunidade no Distrito Federal. Foi investigada a associação de EHNA com síndrome metabólica (SM) e seus componentes individuais.

**TIPO DE ESTUDO E LOCAL:**

Estudo seccional realizado com 139 indivíduos com idade igual ou superior a 55 anos.

**métodos:**

EHNA foi diagnosticada por protocolo clínico para exclusão de indivíduos com sinais de desordens hepáticas, consumo abusivo de álcool e influência hepatotóxica de medicamentos. Fenótipos de EHNA foram classificados com base na ecogenecidade sonográfica. Síndrome metabólica foi definida com base no critério do National Cholesterol Education Program Adult Treatment Panel III. Exames laboratoriais foram realizados para complementar a avaliação clínica bem como diagnosticar SM.

**Resultados:**

EHNA encontrava-se presente em 35,2% dos indivíduos. Em conjunto, os dois fenótipos mais intensos correlacionaram-se com níveis aumentados de glicemia, triglicérides e VLDL lipoproteína (muito baixa densidade) colesterol em jejum. Síndrome metabólica foi diagnosticada em 25,6% dos casos. Além da associação entre EHNA e variáveis discretas representativas da síndrome metabólica, análise não paramétrica confirmou a relação (p < 0,05) entre a manifestação esteatótica e a entidade sindrômica.

**Conclusões:**

Comparado com a literatura, este estudo revelou freqüência de EHNA idiopática em brasileiros idosos e de meia-idade maior que a descrita em outros estudos. Nossos achados sugerem que metabolismo glicêmico anormal conjugado a um maior aporte lipídico e/ou biossíntese exacerbada no fígado são prováveis mecanismos fisiopatogênicos da EHNA na população estudada.

## Introduction

Non-alcoholic fatty liver disease (NAFLD) is a clinicopathological syndrome that ranges from simple steatosis to steatohepatitis, fibrosis or cirrhosis. It is characterized by diffuse or focal fat accumulation in the hepatic parenchyma of patients who deny any history of abusive alcohol consumption.^1^ NAFLD may progress to end-stage liver disease and hepatocellular carcinoma.^2^ It is not possible to predict accurately which patients are at risk of progression and development of associated liver complications, but age is increasingly recognized as a predisposing element.^3^

The onset of the fatty liver phenotype is not well understood, and the leading physiopathological hypothesis links consumption of fat-rich foods and hepatic fat accumulation due to insulin resistance.^2,3^ Insulin resistance, together with obesity, hypertension and dyslipidemia, constitute a state of cardiovascular risk that is defined as metabolic syndrome. The understanding that metabolic disorders are prevalent among the elderly and the lack of reliable estimates in the literature on the prevalence of NAFLD in Brazil are valid justifications for studies on the natural history of the idiopathic steatotic manifestation of NAFLD and its associated metabolic conditions among the elderly population in Brazil.

## OBJECTIVE

The aim of this study was to assess the occurrence of idiopathic NAFLD in a sample of community-dwelling middle-aged and older adults living in the Brazilian Federal District, and to evaluate its association with individual components of metabolic syndrome and with the syndrome as a whole.

## METHODS

The present cross-sectional study was performed using data on 177 people living in urban areas on the outskirts of the Brazilian Federal District who were aged 55 years or older at the time of the study. These individuals were recruited to undergo health screenings at the Universidade Católica de Brasília (UCB) between August 2003 and April 2004. This was a non-probabilistic, convenience sample of consecutive cases.

NAFLD was diagnosed by means of a protocol that brought together clinical, laboratory and ultrasound examinations. Medical examinations were conducted to exclude individuals exhibiting clinical signs of active or past liver infection, inflammation or malignancies; to avoid patients with abusive alcohol consumption on the basis of the history provided; and to rule out any influence from hepatotoxic drugs. On admission, each subject was examined by means of a medical procedure adapted from Bellentani et al.,^4^ which is briefly described in the following: *i*) detailed medical history of any acute hepatitis, surgery and drug or alcohol abuse, and also any history of previous diagnoses of cirrhosis, hemochromatosis or any congenital liver disease; *ii*) careful investigation of any current sign of liver or biliary disease, with emphasis on right upper quadrant pain, jaundice, malaise, anorexia, nausea, vomiting, scleral icterus, palmar erythema, ascites, hepatomegaly, splenomegaly and thrombocytopenia; and *iii*) detailed inventory of drugs in use or used within the last six months prior to the study, with emphasis on drugs with known hepatotoxicity.

The alcohol consumption history, recumbent blood pressure and medications in use were independently assessed by a physician and a nutritionist from their respective interviews with each patient and, whenever possible, these data were checked with family members. These were confirmed at least once by the physician, at a third, independent consultation prior to the ultrasound session. The amounts of alcohol consumed were derived from the history of alcoholic beverages consumed, frequency of drinking per week and volume of drinking per day. Men and women who consumed less than 30 or 20 grams of alcohol/day, respectively, over the last two years were considered to be non-drinkers. The nutritional consultation also had the aim of obtaining the waist circumference, to allow blind identification of individuals with metabolic syndrome, which was defined strictly in accordance with the criteria of the National Cholesterol Education Program Adult Treatment Panel III.^5^

The laboratory tests included serum liver tests (aspartate aminotransferase [AST], alanine aminotransaminase [ALT], gamma-glutamyl transpeptidase [γGT] and alkaline phosphatase [ALP]), hepatitis B serological tests (anti-HBc IgM and IgG), hepatitis C serological tests (antibodies for hepatitis C) and total blood count. Serum fasting glucose (GLU), triglycerides (TGL), total cholesterol (CHL) and fractions (low-density lipoprotein [LDL], very low-density lipoprotein [VLDL] and high-density lipoprotein [HDL]) were also obtained. All the above tests were performed as part of the routine clinical analysis.

**Figure 1:**
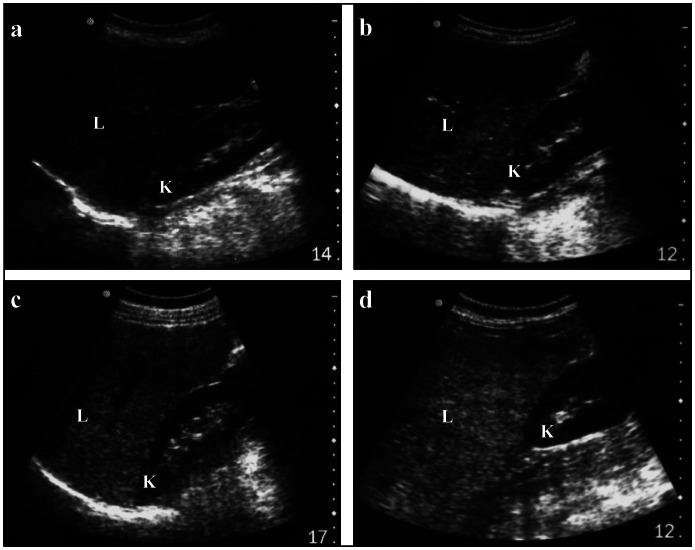
Sagittal ultrasound scans showing echo intensities in both liver parenchyma (L) and renal cortex (K). The panels represent cases in which liver steatosis was not observed (a), mild (b), moderate (c) and severe (d).

Abdominal ultrasound examination was carried out on all patients by one specialist on the same equipment (Sono Site 180), using a convex 3.5 MHz probe. Sagittal hepatic sections encompassing longitudinal images of the right lobe of the liver and the ipsilateral kidney were obtained. Fatty infiltration was graded qualitatively into four classes according to subjective assessment of the contrast between the hepatic parenchyma and the renal cortex, in terms of echo intensity:^6^ non-observed (grade 0), mild steatosis (grade I), moderate steatosis (grade II) and severe steatosis (grade III) (Figure 1). NAFLD was defined as a fatty liver found on ultrasound examination of non-drinkers in the absence of the following: hematological macrocytosis (which would be suggestive of alcohol abuse), consumption of possible hepatotoxic drugs, symptomatic or asymptomatic hepatitis B and C infection, and clinical findings compatible with other liver disorders. No cases of focal fatty infiltration were observed in this study.

This study was approved by the Ethics Committee of the university in which the study was undertaken, and it was conducted in accordance with the provisions of the Helsinki Declaration. Only patients who gave their written consent were included.

The data were expressed as means ± standard deviations (SD). Differences in continuous variables between groups were investigated with multivariate analysis using the Bonferroni correction. Categorical variables were analyzed by the chi-squared and Fisher exact tests. In all cases, p < 0.05 was taken to indicate significance.

## RESULTS

Data from 38 individuals enrolled during the clinical screening were eliminated from the database for the following exclusion reasons: five due to excessive alcohol intake, three due to clinical findings suggestive of liver disease, two due to use of a potentially hepatotoxic drug (methyldopa), one due to confirmed presence of macrocytosis, 12 due to unavailability for laboratory tests and 15 due to reactivity to hepatitis B or C. Thus, 139 subjects fulfilled the inclusion criteria: 20 males and 119 females of mean age 67.0 years (SD = 5.0) and age range from 55 to 85 years. The way in which the sample was constituted probably accounted for the differences regarding age, in which the men were older than the women.

The demographic and laboratory data are shown in Table 1. The ranges of ALT, γGT, AST and ALP were 7-86, 5-91, 11-77 and 101-376 U/l, respectively. Abnormal levels of liver function markers were discovered in 29 patients (20.9%). One patient (0.7%) had abnormal AST alone (> 46 U/l), i.e. presenting normal ALT, ALP and γGT; two patients (1.4%) had abnormal ALT alone (> 50 U/l); seven patients (5.0%) had abnormal ALP alone (> 300 U/l); and thirteen patients (9.4%) had abnormal γGT alone (< 10 U/l: n = 8; > 48 U/l: n = 5). Incidental abnormal ALT (> 50 U/l) together with other markers was observed in two patients with AST elevation (1.4%) and two other patients with γGT elevation (1.4%). Simultaneous abnormal ALP and γGT levels were also detected in two other cases (1.4%). No cases of ALP levels below 70 U/l were observed. These clinical and laboratory results were consistent with a general good state of health among these patients, in terms of gastrointestinal appearance and functions.

**Table 1:** Anthropometric, clinical and laboratory data on the subjects

	Men (n = 20)	Women (n = 119)	All cases (n = 139)	p*
Age (years)	71.0±5.8	66.6±5.7	67.0±5.0	< 0.05
WC (cm)	96.4±7.9	88.1±10.2	89.2±10.3	NS
SAP (mmHg)	144.5±22.0	135.0±17.0	136.4±18.2	NS
DAP (mmHg)	88.0±11.0	83.0±11.0	83.7±11.3	NS
TGL (mg/dl)	138.3±66.7	156.3±68.0	153.7±67.8	NS
CHL (mg/dl)	193.8±29.4	230.5±40.5	225.2±41.1	< 0.001
LDL (mg/dl)	119.5±25.8	142.4±36.6	139.1±36.1	< 0.01
VLDL (mg/dl)	27.8±13.3	30.7±11.8	30.3±12.0	NS
HDL (mg/dl)	46.6±10.2	58.2±13.0	56.5±13.2	< 0.001
GLU (mg/dl)	89.8±22.5	90.5±28.6	90.4±27.7	NS
AST (U/l)	27.0±7.7	26.2±10.0	26.3±9.6	NS
ALT (U/l)	24.2±9.3	21.7±12.4	22.1±12.0	NS
ALP (U/l)	206.2±55.2	196.0±56.8	197.5±56.5	NS
γGT (U/l)	33.2±23.9	21.7±12.0	23.4±14.8	< 0.05

* Comparison between male and female subjects. WC = waist circumference; SAP = systolic arterial pressure; DAP = diastolic arterial pressure; TGL = triglycerides; CHL = total cholesterol; LDL = low density lipoprotein; VLDL = very low density lipoprotein; HDL = high density lipoprotein; GLU = fasting serum glucose; AST = aspartate aminotransferase; ALT = alanine aminotransferase; ALP = alkaline phosphatase; γGT = gamma-glutamyl transpeptidase; NS = non-significant.

NAFLD was found in 35.2% (n = 49) of the 139 subjects. Most of the steatotic patients (n = 25) presented echogenicity compatible with mild fatty accumulation, followed in frequency by the moderate (n = 18) and severe (n = 6) phenotypes. No gender adjustments were made, because reports have usually described similar frequencies among men and women aged 55 years and over.^7,8^

To characterize the metabolic state of the group, the five components of metabolic syndrome were quantified. The vast majority of the patients presented severely elevated serum fat and arterial pressure levels (Table 1). The mean systolic and diastolic pressures were consistent with the observation that 74.8% of all the individuals had at least one abnormal measurement (≥ 130 or ≥ 85 mmHg, respectively). The hyperlipemic profile derived from the fact that respectively 75.5, 58.3 and 38.8% of the samples showed total, LDL and VLDL cholesterol levels above their normal upper limits (> 200, > 130 and > 30 mg/dl). Accordingly, 40.3% of the subjects presented hypertriglyceridemia (≥ 150 mg/dl), whereas HDL cholesterol < 40 mg/dl for males and < 50 mg/dl for females (i.e. below the recommended levels) was observed in 24.5% of the group. Waist circumference exceeded the threshold for abdominal obesity (> 102 cm for males and > 88 cm for females) in 38.1% of the patients. Only a small number of the patients (10.8%) had glycemia levels ≥ 110 mg/dl, i.e. above normal values. After age-adjustment analysis, none of the means showed significant changes.

Taking into account that four out of the five criteria for metabolic syndrome (fasting glucose, triglycerides, arterial pressure and waist circumference) were not influenced by gender in our analysis (Table 1), the whole group was considered together in estimating the frequency of metabolic syndrome. Under our conditions, roughly one in every four of these middle-aged and older adults (25.9%) was diagnosed as presenting metabolic syndrome (i.e. three or more of the criteria fulfilled). Four criteria were found in 11.5% of our subjects, while all five criteria were fulfilled in 2.9%. Only 10.8% of the patients did not satisfy any of the diagnostic criteria.

Taken together, the two most intense manifestations of NAFLD (II-III) presented higher levels of serum fasting glucose, total triglycerides and VLDL cholesterol than did the non-steatotic group (Table 2). It is important to draw attention to the fact that these serum metabolites were strong markers for metabolic syndrome, and that the non-parametric results confirmed an association (p < 0.05) between the steatotic manifestation and the occurrence of the syndrome itself (Table 3). This latter association can be illustrated by the fact that subjects with metabolic syndrome presented a ratio of steatotic to non-steatotic cases that was more than twice as large (ratio = 1.0) as the ratio presented by subjects without the syndrome (ratio = 0.4). No liver function markers showed predictive value for any of the fatty liver phenotypes.

**Table 2:** Mean anthropometric and clinical features, and mean levels of metabolic and liver function markers according to the stage of the non-alcoholic fatty liver disease (NAFLD)

	NAFLD phenotype		
	0 (n = 90)	I (n= 25)	II-III (n = 24)
Age (years-old)	67.7±5.4	65.5±5.8	65.7±5.2
WC (cm)	88.1±9.9	92.8±10.7	89.4±11.0
SAP (mmHg)	135.8±17.9	137.4±14.9	137.5±22.7
DAP (mmHg)	83.4±11.9	84.4±9.2	84.2±11.2
GLU (mg/dl)	87.2±25.1	90.6±21.9	102.0±38.8*
CHL (mg/dl)	223.8±44.5	224.0±31.4	231.9±37.2
LDL (mg/dl)	140.0±39.5	134.8±27.6	140.5±30.9
VLDL (mg/dl)	27.9±10.4	34.4±15.2*	35.0±12.0*
HDL (mg/dl)	56.9±13.6	54.7±11.2	57.0±14.1
TGL (mg/dl)	139.7±52.1	171.8±76.0	187.6±93.6†
AST (U/l)	26.6±9.3	24.8±6.6	27.0±13.2
ALT (U/l)	21.9±11.9	21.0±6.5	23.7±16.5
ALP (U/l)	203.5±56.0	181.5±55.5	191.5±57.5
γGT (U/l)	23.3±16.3	20.8±8.0	26.2±14.4

* p < 0.05 when compared with the non-steatotic (0) group;

† p < 0.01 when compared with the non-steatotic (0) group. WC = waist circumference; SAP = systolic arterial pressure; DAP = diastolic arterial pressure; GLU = fasting serum glucose; CHL = total cholesterol; LDL = low density lipoprotein; VLDL = very low density lipoprotein; HDL = high density lipoprotein; TGL = triglycerides; AST = aspartate aminotransferase; ALT = alanine aminotransferase; ALP = alkaline phosphatase; γGT = gamma-glutamyl transpeptidase.

**Table 3:** Comparison of frequencies of non-alcoholic fatty liver disease (NAFLD) phenotypes between subjects with and without metabolic syndrome. Observed and predicted frequencies are displayed together with percentages (between parentheses), for each group

			With metabolic syndrome n = 36	Without metabolic syndrome n = 103	p*
NAFLD frequencies	0	count	18 (50.0)	72 (69.9)	< 0.05
		expected count	23.3 (64.7)	66.7 (64.7)	
	I	count	11 (30.6)	14 (13.6)	
		expected count	6.5 (18.1)	18.5 (18.0)	
	II-	count	7 (19.4)	17 (16.5)	
	III	expected count	6.2 (17.2)	17.8 (17.3)	

* χ^2^ = 6.03; degrees of freedom (df) = 2.

## DISCUSSION

The main finding from this study was that approximately one in every three of these Brazilian middle-aged and older adults enrolled in the study presented findings compatible with cryptogenic NAFLD. Moreover, the present report indicates a prevalence of NAFLD that is notably higher than what was derived from studies carried out elsewhere on younger cohorts. Compared with data in the literature, the present study suggests that the frequency of idiopathic NAFLD among these healthy, predominantly elderly Brazilians was greater than among healthy, predominantly middle-aged Korean,^7^ Japanese^9^ and Italian^10^ cohorts. Studies conducted in populations with westernized lifestyles have found prevalence of liver steatosis of approximately 20%.^1,11,12^ This greater prevalence corroborates other reports^4,7,10^ in which greater age is described as an important risk factor for fatty liver disease, and it may indicate that the social and health conditions inherent to developing countries intensify the frequency of NAFLD. In addition, these results allow the elderly to be included in the general statement that liver steatosis is frequent even in the absence of other data suggestive of hepatic disorders.^10,13^

The presence of NAFLD was significantly associated with a spectrum of findings compatible with impaired insulin action. A series of physiopathological, clinical and laboratory investigations have supported the notion that insulin resistance has a central role in the pathogenesis of NAFLD^14-16^. In the present report, states of insulin resistance were expressed by means of fasting glucose levels. This is a rather crude indicator for insulin action, but it is of practical use for large surveys. Moreover, NAFLD has also been shown to correlate with the heterogeneous condition known as metabolic syndrome, which is broadly related to insulin resistance. Despite correlation with the syndrome as a whole, the present results most likely underscore the actual level of associations between the fatty liver phenotype and each component of metabolic syndrome, since this study was not designed to exclude subjects undergoing cardiovascular, hypolipemic and hypoglycemic pharmacotherapy.

It is likely that the high visceral fat content observed, which was reflected by the proportion of individuals with waist circumference measurements greater than the acceptable limits (almost 40% of the subjects), acted as a predisposing element in the development of NAFLD, as described elsewhere.^17^ Therefore, another contribution from the present report consists of emphasizing that the association between NAFLD and features of metabolic syndrome may be extended to the whole Brazilian population. Moreover, these findings indicate that increased fat delivery and/or sustained endogenous biosynthesis in the liver are the most likely physiopathogenic mechanisms underlying the onset of NAFLD under our conditions, to the detriment of the hypothesis of diminished lipoprotein secretion.^18^ This is because an accumulation of deep abdominal fat may lead to increased delivery of fatty acids to the liver, followed by increased synthesis and secretion rates for triglycerides and VLDL particles, and by the onset of liver steatosis in susceptible individuals. These hypotheses need to take into account human genomic and nutritional heterogeneity, which deserve consideration.

The major criticisms that may be made of the present study probably lie in the impossibility of ruling out covert or intermittent alcohol consumption, or excessive liver echogenicity unrelated to fatty infiltration. Nonetheless, the absence of clinical and biochemical evidence for alcohol abuse among the subjects selected lessened the possibility of recent or chronic alcohol use. Moreover, in alcoholic liver disease, abstinence usually leads to rapid resolution of the fatty infiltrate.^19^ In addition, screening for reactivity to hepatitis B and C probably prevented the inclusion of individuals presenting cirrhotic conditions. Because of the costs involved in this type of survey, it was not possible to enlarge the sample used in the study. Nonetheless, since most of Brazil's aging and elderly population share similar socioeconomic standards, characterized by low income, low schooling levels and poor nutritional conditions,^20-22^ we would tend towards considering our findings to be illustrative of the scenario in this country.

Brazil is a developing country in which the efforts to evaluate the prevalence of and burden caused by liver conditions may be considered insufficient. To the authors’ knowledge, there is a lack of systematic assessments of NAFLD on either a local or a national scale. This is the first report aimed at evaluating the occurrence of such conditions among the aging population of Brazil.

## CONCLUSIONS

Taken together, our results indicate that the prevalence of NAFLD among Brazilians aged 55 years and over is greater than what is seen elsewhere. Also, our findings implicated metabolic disorders that are compatible with metabolic syndrome presenting the steatotic phenotype. The present study is a report from an ongoing prospective study on the outcomes from dietary pattern and associated metabolic disorders, with regard to health status within the aging population of Brazil.
